# Facilitated early discharge in Wandsworth

**DOI:** 10.1192/bjo.2021.336

**Published:** 2021-06-18

**Authors:** Mudasir Firdosi, Allerdiena Hubbeling, Twaisha Kapoor

**Affiliations:** St Georges University of London

## Abstract

**Objective:**

There is limited research surrounding facilitated early discharge (FD) and Home Treatment Teams (HTTs). This study aimed to compare patients who received FD with patients who were discharged without FD to identify whether there were significant differences in terms of social demographics, illness characteristics, health outcome and treatment duration. Using this data we furthermore aimed to provide proposals to help advance the effectiveness of FD, as well as suggesting concepts of where future research should lie.

**Case report:**

A randomised sample of patients who received FD and patients who were discharged without FD was obtained from a South London Hospital. This was manually narrowed down to patients specifically treated by the Wandsworth Home Treatment Team (WHTT). Socio-demographic and clinical data were then attained from the patients’ electronic records to compare and statistically analyse between the two groups.

**Discussion:**

Patients who received FD from the WHTT were found to have significantly less previous psychiatric admissions compared to those who were discharged without FD (p = 0.032). All other variables were found to have no association with FD.

**Conclusion:**

Having a high number of previous psychiatric admissions seems to be an aspect that decreases the chance of being allocated FD. This variable can be seen as an indicator of severity of illness and a challenging social environment; it could therefore be valuable to take this variable into consideration when allocating FD. Furthermore, total treatment duration was found to not be significantly different for FD and non-FD patients, thus supporting the use of CRHTTs as an equivalent alternative for inpatient admission, however, national scale research should be conducted to strengthen and expand on these findings.

